# Halophilic archaeon *Halorhabdus rudnickae* enhances epithelial barrier integrity in Calu-3 cells

**DOI:** 10.3389/fcimb.2026.1976735

**Published:** 2026-10-29

**Authors:** Krzysztof Krawczyk, Serafin Zawadzki, Sylwia Michlewska, Sebastian Wawrocki, Aleksandra Roszko, Joanna Wieczfinska, Magdalena Kowalewicz-Kulbat

**Affiliations:** 1 Faculty of Biology and Environmental Protection, Department of Immunology and Infectious Biology, Institute of Microbiology, Biotechnology and Immunology, University of Lodz, Lodz, Poland; 2 Faculty of Biology and Environmental Protection, Department of General Biophysics, University of Łódź, Łódź, Poland; 3 BioMedChem Doctoral School of the University of Łódź and Łódź Institutes of the Polish Academy of Sciences, Łódź, Poland; 4 Faculty of Biology and Environmental Protection, Laboratory of Microscopic Imaging and Specialized Biological Techniques, University of Lodz, Lodz, Poland; 5 Centre of Regenerative Medicine, Medical University of Bialystok, Bialystok, Poland; 6 Department of Immunopathology, Medical University of Lodz, Lodz, Poland

**Keywords:** air-liquid interface (ALI), airway epithelial barrier, Calu-3 cells, halophilic archaea, *Halorhabdus rudnickae*

## Abstract

Halophilic archaea are a distinct domain of life, separate from Bacteria and Eukarya, that thrive in highly saline environments, including salt mines. Their impact on the human immune system is poorly known. The respiratory epithelium is the first line of defence against inhaled environmental agents, including pollutants, allergens and microorganisms. Disruption of epithelial barrier integrity is linked to the pathogenesis of allergic and inflammatory airway diseases. In this study we examined the interaction of *Halorhabdus rudnickae* WSM-64^T^, isolated from the Barycz area of the Wieliczka Salt-Mine in Poland with the human bronchial epithelial cell line Calu-3. We found that this halophilic archaeal strain did not cause cytotoxic or genotoxic effects on Calu-3 cells and significantly enhanced the healing process of physically damaged monolayers of cells compared to unstimulated cells. Moreover, halophilic archaeal stimulation of Calu-3 was associated with significantly increased fluorescence intensity of ZO-1, scaffolding protein responsible for organizing the junctional proteins and stabilising cell barriers. Exposure to *Hrd. rudnickae* significantly decreased the secretion of TGF-beta by Calu-3 cells, suggesting a potential ability to counteract the pathological changes driven by TGF-beta. In the air-liquid interface model we observed significant reduction in FITC-dextran permeability, indicating improved barrier integrity after stimulation of Calu-3 with halophilic archaea. Our findings thus show that halophilic archaeon *Hrd. rudnickae* isolated from a Polish salt mine can induce the enhancement of epithelial barrier integrity in the Calu-3 cells, providing a conceptual framework for future immunomodulatory strategies in patients with pulmonary allergic disorders.

## 1 Introduction

Respiratory epithelial cells are nonhematopoietic cells playing a crucial role in orchestrating the host immune response. These cells are in constant contact with the external environment, which makes them the main barrier separating the body’s interior from environmental threats. An efficiently functioning epithelial barrier consists of three components: cilia and mucus that remove physical threats, intercellular tight and adherens junctions that regulate paracellular transport of substances, and antimicrobial factors that kill inhaled pathogens (Ganesan et al., 2013). In recent years, the epithelial barrier hypothesis has been formulated, based on the observation that the damage of the epithelial barrier is noted in many chronic diseases, including atopic dermatitis, asthma, allergic rhinitis, inflammatory bowel disease and coeliac disease (Akdis, 2021; Clemente et al., 2003; De Benedetto et al., 2011; Steelant et al., 2016; Sun et al., 2024; Toedter et al., 2012; Xiao et al., 2011). Damage to epithelial cells has been linked to a disruption to the epithelial barrier’s integrity, the release of alarmins (IL-25, IL-33, and TSLP) and the development of inflammation. This, in turn, has been linked to further disruption of the epithelial barrier and, consequently, the penetration of harmful environmental factors into the tissues (Pat et al., 2024; Saito et al., 2023; Zeyneloglu et al., 2025).

There are many factors, like ozone, nitrogen dioxide, surfactants or emulsifiers, present in processed foods, house dust mite and pollen allergens, as well as cytokines (including IL-4 and IL-13), which may have a negative impact on the structure of the epithelial barrier (Mitamura et al., 2021). The mechanisms responsible for the damage to the epithelial barrier include: impaired expression and degradation of tight junctions (TJs); production of reactive oxygen species that oxidise cellular structures; induction of inflammation and activation of pathways responsible for cell death (Pat et al., 2024). So far, there are no pharmaceutical agents with proved regenerative potential for the epithelial barrier, thus there is ongoing research to identify novel pro-regenerative factors involving the use of microorganisms secreting various metabolites with therapeutic potential.

*Archaea* constitute one of the three domains of life differing significantly from *Bacteria* and *Eukarya*, which are often identified as extremophiles able to survive in extremely unfavourable conditions (low/high pH, low/high temperature, high salinity or the presence of heavy metals) (Baker et al., 2020; Cavicchioli, 2011). They represent a group of microorganisms that have been identified as having biotechnological and medical potential (Adamiak et al., 2021; Corral et al., 2019; Pfeifer et al., 2021). Halophilic archaea are microorganisms which inhabit highly saline environments, like brines, rock salt, salt lakes and salt mines (Puławska et al., 2025; Stan-Lotter and Fendrihan, 2015). So far no species of halophilic archaea have been identified that could be regarded as a direct human pathogens (Duller and Moissl-Eichinger, 2024).

In our recent study, we have identified the airborne halophilic archaea at the Bochnia Salt Mine in the winter (*Halalcalicoccus subterraneus*, *Halococcus hamelinensis*) and in the summer season (*Halococcus salifodinae, Natrinema versiforme, Halococcus morrhuae, Halalkalicoccus paucihalophilus, Halococcus dombrowskii, Halalkalicoccus subterraneus, Halococcus hamelinensis*), and we found the seasonal fluctuations and variability under the influence of physical-chemical conditions (Puławska et al., 2025, 2026).

Previously, we have shown that two halophilic archaea, *Halorhabdus rudnickae* and *Natrinema salaciae*, can enter human monocyte-derived dendritic cells and stimulate the expression of surface receptors CD83, CD86, CD80 (involved in immune synapse DC-T cell signalling), the production of cytokines by DCs (IL-10, IL-12p40, TNF-alpha) and by autologous T lymphocytes (IFN-gamma, IL-10, TNF-alpha, IL-12, IL-13) (Krawczyk et al., 2022). Moreover, no apoptotic/necrotic effect under stimulation with halophilic archaea was observed in DCs (Krawczyk et al., 2025). The protective effect of halophilic archaea from DNA damage caused by *Staphylococcus aureus* Enterotoxin type B (SEB) (Krawczyk et al., 2025) has been described. However, the interaction between halophilic archaea and epithelial cells remains poorly understood.

Thus, in this study we examined the interaction of *Hrd. rudnickae* WSM-64^T^ (= DSM 29498T; = CECT 8673T) strain, isolated from the Barycz mining area of the Wieliczka Salt-Mine in Poland (Albuquerque et al., 2016) with human epithelial cells using the Calu-3 cell line as an *in vitro* model. We observed that *Hrd. rudnickae* did not induce any cytotoxic or genotoxic effects on epithelial Calu-3 cell line, and at the same time appears to strengthen the epithelial barrier in both monolayer and air-liquid interface (ALI) models.

## 2 Materials and methods

### 2.1 Calu-3 culture

The human airway epithelial cell line Calu-3 was obtained from LGC Standards (American Type Culture Collection, Cat. No. ATCC-HTB-55) and cultured according to the protocol described by Braakhuis et al (Braakhuis et al., 2020). Briefly, the Calu-3 cells were grown in a complete medium (c-MEM): Minimum Essential Medium (MEM, GlutaMAX™ Supplement; ThermoFisher, 41090028), 10% FBS (BioWest, S181A), 1% Penicillin/Streptomycin (Penicillin–Streptomycin Solution 100X; BioWest, L0022) and 1% Non-Essential Amino Acids (MEM Non-Essential Amino Acid Solution 100X; ThermoFisher, 11140050), in a humidified atmosphere at 37 °C with 5% CO_2_ in 75 cm² tissue culture flasks (Googlab Scientific, G77080033).

The cells were passaged at least twice after thawing, prior to use in experiments. The cells were observed under a light microscope and were passaged every 3–4 days when they reached 70–90% confluency. Calu-3 cells were washed twice with Dulbecco’s phosphate-buffered saline (DPBS) (without calcium and magnesium; Biowest, L0615), and then trypsinised with 0.25% trypsin-EDTA for 5 minutes (Biowest, LO931). All experiments were performed using passages 8–20.

### 2.2 Culture of halophilic archaea

Halophilic archaeal strain, *Halorhabdus rudnickae* (WSM-64^T^=DSM29498^T^), was kindly provided by Dr Luciana Albuquerque (Center for Neuroscience and Cell Biology, Biotech, Biocant Park, Cantanhede, Portugal) (Albuquerque et al., 2016). Halophilic archaea were cultivated in 100 mL of liquid *Halobacteria* medium (HBM) (5 g/L yeast extract, 5 g/L casamino acids, 1 g/L Na-glutamate, 2 g/L KCl, 3 g/L Na_3_-citrate, 20 g/L MgSO_4_ × 7 H_2_O, 36 mg/L FeCl_2_ × 4 H_2_O, 360 ng/L MnCl_2_ × 4 H_2_O, 200 g/L NaCl), as described previously (Krawczyk et al., 2022) and harvested from 48-hour cultures in the logarithmic growth phase, as previously described (Krawczyk et al., 2025, 2022). *Hrd. rudnickae* were centrifuged at 4 °C for 15 minutes at 4500 × g. The pellets were transferred to new 15 mL tubes and washed by resuspension in 10 mL of 4 °C DPBS, then centrifuged at 4 °C for 15 minutes at 4500 × g. The cells were resuspended in c-MEM medium immediately prior to stimulation. All experiments were performed using MOI 10 (Calu-3: *Hrd. rudnickae*; 1:10).

### 2.3 Viability assay with CCK-8 kit

To assess the potential cytotoxicity of *Hrd. rudnickae* on Calu-3 cells the colorimetric assay Cell Counting Kit-8 (CCK-8; Sigma-Aldrich, 96992) was performed as described previously (Macieja et al., 2023). The Calu-3 cells were collected and counted, then seeded at a density of 1x10^4^ cells per well in a 96-well tissue culture plate (TPP^®^, flat-bottom; Merck, Z707902) in c-MEM for 24 hours prior to the experiment. The cells were stimulated with c-MEM with 1x10^5^ *Hrd. rudnickae* or with c-MEM with 10% DMSO (Dimethyl sulfoxide; Sigma-Aldrich, D5879) as a positive control. Calu-3 cells stimulated with c-MEM were used as a negative control. After 24 and 48 hours, the Calu-3 cells were washed with 37 °C DPBS and supplemented with 37 °C c-MEM containing a 10% CCK-8 solution. After 3 hours of incubation, the absorbance was measured at 450 nm using a SpectraMax i3 (Molecular Devices). Cell viability was determined by comparison with the negative control which was 100%.

### 2.4 Comet assay – detection of DNA damage

An alkaline comet assay was performed to assess the level of potential DNA damage induced by *Hrd. rudnickae* in Calu-3 cells following the protocols described by Collins AR (Collins, 2004) and Macieja et al (Macieja et al., 2023). Calu-3 cells were collected and counted, then seeded at a density of 5x10^4^ cells/well in a 48-well culture plate, 24 hours prior the stimulation. To determine the level of DNA damage, the Calu-3 cells were stimulated with either c-MEM (negative control) or c-MEM containing 5x10^5^ *Hrd. rudnickae*. After 24 and 48 hours, the Calu-3 cells were washed with 37 °C DPBS and trypsinised with 0.25% trypsin-EDTA for 5 minutes. Additionally, a separate population of unstimulated cells that had been cultured for 24 or 48 hours were treated with 50 µM tert-butyl hydroperoxide (TBH, Sigma-Aldrich) in c-MEM and stored for 15 minutes on ice as a positive control. The cells were suspended in 0.75% low-melting-point (LMP) agarose (Sigma-Aldrich) and placed on microscope slides that had been precoated with 0.5% normal-melting-point (NMP) agarose. The slides were placed in lysis buffer ((2.5 M NaCl, 100 mM EDTA, 10 mM Tris and 1% Triton X-100 (v/v); Sigma-Aldrich)) and incubated overnight at 4 °C. The slides were transferred to an unwinding buffer appropriate for the alkaline version of the Comet assay for 20 minutes. Electrophoresis was performed at 17 V and 32 mA for 20 minutes. The slides were dried and prepared for analysis by staining with DAPI (5 µg/mL) (Sigma-Aldrich, D9542).

Comet assay analysis was performed using an Eclipse fluorescence microscope (Nikon, Tokyo, Japan), which was attached to a COHU 4910 video camera (COHU, Poway, CA, USA). The Lucia Comet 4.51 computer-based image analysis system (Laboratory Imaging, Prague, Czech Republic) was used for this analysis. The microscope was equipped with a UV filter block consisting of an excitation filter with a wavelength of 359 nm and a barrier filter with a wavelength of 461 nm.

### 2.5 Wound healing assay

The scratch assay (also known as the wound healing assay) is an *in vitro* method that allows cell migration and/or proliferation to be studied after the disruption of a cell monolayer (Molefe et al., 2026). A scratch assay was conducted to study whether *Hrd. rudnickae* had an impact on human epithelial cell recovery. Calu-3 cells were seeded in a 96-well plate at a density of 2.5x10^4^ cells per well. After incubating the cells for 30 minutes at room temperature to allow them to attach to the bottom of the wells, the plates were transferred to the xCELLigence RTCA eSight system, which was housed in a humidified incubator maintained at 37 °C and 5% CO_2_. After 24 hours, once a confluent monolayer had been established, a uniform scratch was made in each well using the AccuWound 96 tool (ACEA Biosciences, USA). The wells were then washed with DPBS to remove cell debris and 2.5x10^5^ *Hrd. rudnickae* were added. Data were collected at two-hourly intervals over a 72-hour period and analysed using RTCA eSight software. The results were expressed as a percentage of the wound area covered by cells.

### 2.6 Confocal microscopy

Epithelial cells are connected by tight junctions, specialized structures that form a paracellular barrier to water and solutes, as well as maintaining adhesion between cells (Citi et al., 2024). ZO-1 staining was performed to measure the effect of *Hrd. rudnickae* on the tight junction structure of Calu-3 cells. The cells were seeded at a density of 1x10^4^ cells per well in a µ-Slide 18 Well plate (Ibidi, 81816). After 24 hours, the medium was removed and the cells were stimulated with c-MEM containing 1x10^5^ *Hrd. rudnickae* or c-MEM containing 50 ng/mL IFN-gamma as a disrupting factor (ThermoFisher, RIFNG50). Calu-3 cells in c-MEM were used as a negative control. After 24, 48 and 72 hours, the cells were washed with DPBS, fixed for 15 minutes with 4% formaldehyde (Sigma-Aldrich, 252549) and washed twice with DPBS. The cells were permeabilised with detergent (0.1% Triton X-100/DPBS) for 5 minutes and blocked with 10% skimmed milk in 1% BSA/DPBS for 30 minutes at room temperature. The Calu-3 cells were stained with a ZO-1 antibody (ZO-1 Monoclonal Antibody (ZO1-1A12), Alexa Fluor™ 594, ThermoFisher, 339194FD4) was diluted 1:100 in 1% BSA/DPBS for 1 hour at room temperature in the dark. The cells were mounted using a Leica CV mount (Leica Biosystems, 14046430011) with DAPI (5 µg/mL) (Sigma-Aldrich, D9542). Confocal laser scanning microscopy was performed on a Leica TCS SP8 (Leica Microsystems, Germany) equipped with an HC PL APO CS2 ×63/1.40 oil-immersion objective. Nuclei (DAPI) were excited at 405 nm and detected at 430–480 nm; ZO-1 labelled with Alexa Fluor™ 594 was excited at 594 nm and detected at 610–650 nm. Images were acquired and quantified in Leica Application Suite X (LAS X, Leica Microsystems, Germany). Integrated fluorescence intensity within regions of interest (ROIs) was calculated as the sum of pixel intensities and reported in arbitrary units (a.u.).

### 2.7 Cytokine production

The Calu-3 cells were seeded at a density of 2x10^5^ cells per well in a 24-well plate and left for 24 hours to attach. After 24 hours, the c-MEM was replaced with fresh c-MEM (negative control) and c-MEM containing 2x10^6^ *Hrd. rudnickae*. The supernatants were collected after 24 and 48 hours, centrifuged (4 °C, 10 minutes at 1000 × g) and stored at -80 °C until the assay was performed. The level of TGF-beta, IL-6 and TNF-alpha in culture supernatant of *Hrd. rudnickae*-stimulated Calu-3 cells was determined using the Human DuoSet ELISA (R&D Systems; DY240, DY206, DY210, respectively), following the manufacturer’s instructions. TGF-beta samples were activated using Sample Activation Kit 1 (R&D Systems, DY010). The plates were read using a Varioskan Lux (Thermo Fisher Scientific) set to 450 nm and the results were calculated relative to the generated standard curves. The standard ranges for cytokines were: TGF-beta - 31.2–2000 pg/mL; IL-6 - 9.38–600 pg/mL; TNF-alpha - 15.6–1000 pg/mL.

### 2.8 Air-liquid interface culture

Calu-3 cells were grown in an air-liquid interface (ALI) as described by Braakhuis et al (Braakhuis et al., 2020). The 6-well inserts (Corning^®^ 24 mm Transwell^®^ with a 0.4 µm pore polyester membrane insert; 3450) were apically coated with 10 µg/cm^2^ of collagen I (rat tail; ThermoFisher, A1048301) in DPBS overnight at 37 °C in a humidified atmosphere with 5% CO_2_. The collagen solution was then removed, and the Calu-3 cells were seeded at a density of 5x10^5^ cells/insert in 2 mL of c-MEM in the apical part of the insert, while the basolateral part was filled with 3 mL of c-MEM. After two days, the medium was replaced in the apical and basolateral parts. After an additional two days, the medium in the apical part was removed and the medium in the basolateral part was changed every second day.

The Calu-3 cells in the 14-day ALI culture were supplemented with fresh c-MEM in the basolateral compartment. The apical part was filled with c-MEM (negative control), c-MEM containing 5x10^6^ *Hrd. rudnickae/*insert, or c-MEM containing 50 ng/mL IFN-gamma as disrupting factor (ThermoFisher, RIFNG50).

### 2.9 Transepithelial electrical resistance and paracellular flux measurement

The transepithelial electrical resistance (TEER) of the Calu-3 ALI cultures was measured using a Millicell ERS-2 Voltohmmeter (Merck Millipore, MERS00002), according to the manufacturer’s instructions. The resistance of the Calu-3 cultures was measured, as well as the resistance of the empty inserts (blank). TEER results were obtained by the subtraction of the empty insert values from the measured values and the subsequent multiplication of the result by the cell growth surface area (4.67 cm^2^), according to the equation:


$$\text{TEER}(\text{Ω}\times {\text{cm}}^{2})=({\text{R}}_{\text{sample}}-{\text{R}}_{\text{blank}})\times \text{Area}(4.67\;{\text{cm}}^{2})$$

TEER results were presented as Ω × cm². TEER measurements were performed at 0, 24, 48 and 72 hours after apical stimulation.

Paracellular flux measurements were performed at 48 and 72 hours of stimulation. Paracellular permeability was evaluated by removing the c-MEM from the apical parts, transferring the inserts to a new 6-well plate, and adding the c-MEM to the basolateral part. c-MEM containing 1 mg/mL of fluorescein isothiocyanate (FITC)-dextran 4kDa (Sigma-Aldrich, FD4) was then added to the apical parts of the inserts. The cells were incubated for 4 hours in darkness at 37 °C in a humidified atmosphere with 5% CO_2_. The medium from the basolateral part was collected, and the FITC-dextran fluorescence was determined using a fluorescence reader (SpectraMax i3, Molecular Devices) at 480 nm. Paracellular flux was determined by comparing the results with those of the unstimulated control group.

### 2.10 Actin staining

The Calu-3 ALI culture inserts were washed with DPBS, fixed for 15 min in 4% formaldehyde (Sigma-Aldrich, 252549), and washed twice with DPBS. Actin was stained with Texas Red™-X Phalloidin (Thermo Fisher Scientific, T7471) according to the manufacturer’s instructions (Linke et al., 1999).

Cells were mounted in Leica CV Mount (Leica Biosystems, 14046430011) containing DAPI (5 µg/mL; Sigma-Aldrich, D9542). Confocal laser scanning microscopy was performed on a Leica TCS SP8 (Leica Microsystems, Germany) equipped with an HC PL APO CS2 ×63/1.40 oil-immersion objective. DAPI was excited at 405 nm with emission detected at 430–480 nm, and Texas Red™-X Phalloidin was excited at 594–595 nm with emission detected at 610–640 nm. Image acquisition and visualization were carried out in Leica Application Suite X (LAS X, Leica Microsystems, Germany).

### 2.11 Statistical analysis

Statistical analyses were performed with the GraphPad Prism 8.1 and STATISTICA 12.0 PL program. Data are expressed as mean ± standard deviation. After verifying the types, numbers of data and data assumption by normality Kolmogorov-Smirnov test, non-parametric statistics were used. Differences between more than two groups were analyzed by Kruskal-Wallis non-parametric test, while for comparisons between two groups of impaired data the Mann-Whitney U test was used. p values ≤ 0.05 were considered significant. Unless stated otherwise, the results are presented in the form of a scatter plot, where each point corresponds to a single independent experiment.

## 3 Results

### 3.1 *Hrd. rudnickae* does not induce cytotoxicity or detectable DNA damage in Calu-3 cell line

To determine the cytotoxic effect of halophilic archaea on epithelial cells, the Calu-3 cell line was stimulated with *Hrd. rudnickae* for 24 and 48 hours and analysed using a CCK-8 assay. In this test WST-8 ((2-(2-methoxy-4-nitrophenyl)-3-(4-nitrophenyl)-5-(2,4-disulfophenyl)-2H-tetrazolium, monosodium salt) is reduced by cell dehydrogenases to produce a yellow-coloured product (formazan), the amount of which is directly proportional to the number of living cells. No significant difference in viability was observed between *Hrd. rudnickae*-stimulated Calu-3 cells and unstimulated cells at 24 and 48 hours (**Figure 1A**). The viability of Calu-3 cells stimulated with 10% DMSO (positive control) in c-MEM was significantly lower after 24 and 48 hours compared to unstimulated cells (*p = 0.0022*) (**Figure 1A**).

**Figure 1:**
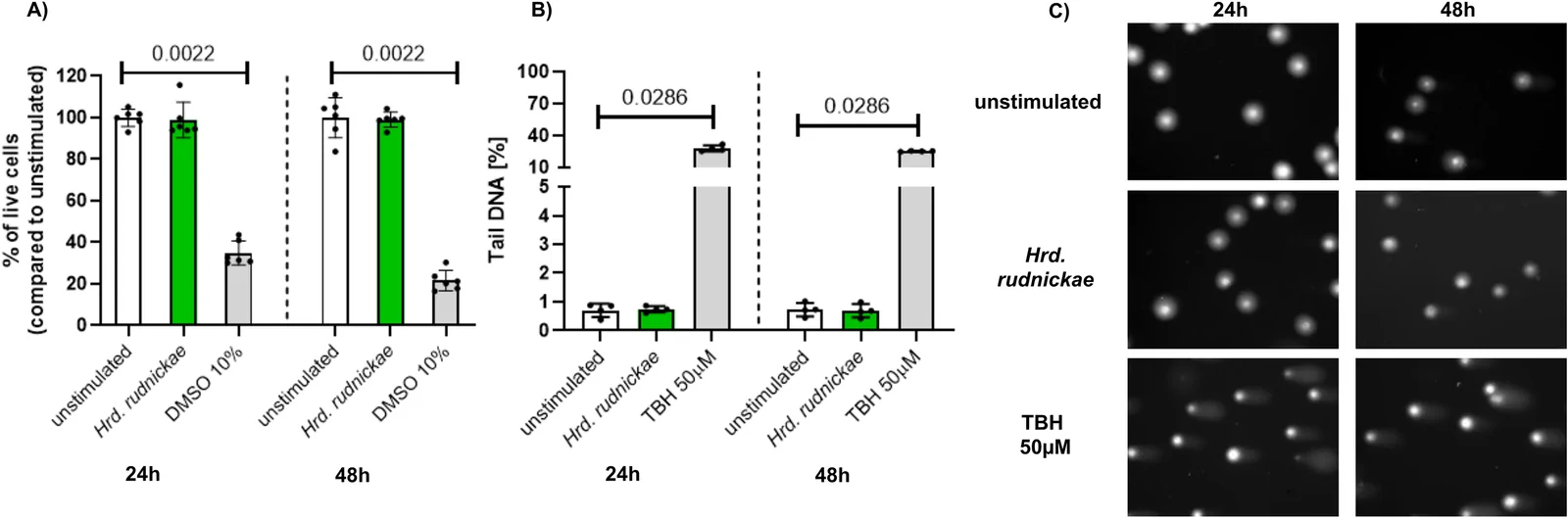
Cytotoxicity and genotoxicity assays of Calu-3 epithelial cells stimulated with *Hrd. rudnickae*. **(A)** Unstimulated Calu-3 cells were used as a negative control. Calu-3 cells were stimulated with *Hrd. rudnickae* or 10% DMSO (positive control) for 24 or 48 hours, and analysed using a CCK-8 assay. Data are shown as a percentage of live cells compared to unstimulated cells. Data are presented as means ± SD, while the dots represent individual results. n = 6, Significant p-values are shown on the graph. **(B)** Unstimulated Calu-3 cells were used as a negative control. Calu-3 cells were stimulated with *Hrd. rudnickae* or with 50 µM TBH (positive control). After 24 and 48 hours, the cells were analysed using an alkaline version of the comet assay. Data are shown as the percentage of tail DNA of the comets. Data are presented as means ± SD, while the dots represent individual results. n = 4. Significant p-values are shown on the graph. **(C)** Representative microscopic images showing the comet assay for unstimulated Calu-3 cells (negative control), cells stimulated with *Hrd. rudnickae*, and cells stimulated with 50 µM TBH (positive control). Cell DNA is represented in white. The solid sphere shows undamaged DNA in the cell nucleus, while the transparent smear (tail) represents damaged DNA. TBH, tert-butyl hydroperoxide; DMSO, dimethyl sulfoxide.

To further analyse whether *Hrd. rudnickae* will not cause any genotoxic effect manifested with DNA breaks in Calu-3 cells an alkaline version of the comet assay was performed. After 24 and 48 hours of stimulation with *Hrd. rudnickae*, no significant changes in the percentage of tail DNA were observed in Calu-3 cells (**Figures 1B, C**), which confirmed the lack of a genotoxic effect of *Hrd. rudnickae* on the Calu-3 cell line. Stimulation with tert-butyl hydroperoxide (TBH; 50 µM, positive control) showed a significant increase in DNA damage, as indicated by a higher amount of tail DNA after 24 *(p = 0.0286*) and 48 hours (*p = 0.0286*) of stimulation. (**Figures 1B, C**).

### 3.2 Cytokine production by Calu-3 cells in response to *Hrd. rudnickae* stimulation

To examine the cytokine response in *Hrd. rudnickae*-stimulated Calu-3 culture supernatants were collected after 24 and 48 hours of stimulation and the levels of IL-6, TNF-alpha and TGF-beta were measured.

Among the analytes tested, only TGF-beta was detectable in the culture supernatants and its level was significantly lower after 24h (*p = 0.0079*) and 48h (*p = 0.0079*), compared to unstimulated cells (**Figure 2**). There was no production of IL-6 and TNF-alpha in culture supernatants (data not shown).

**Figure 2:**
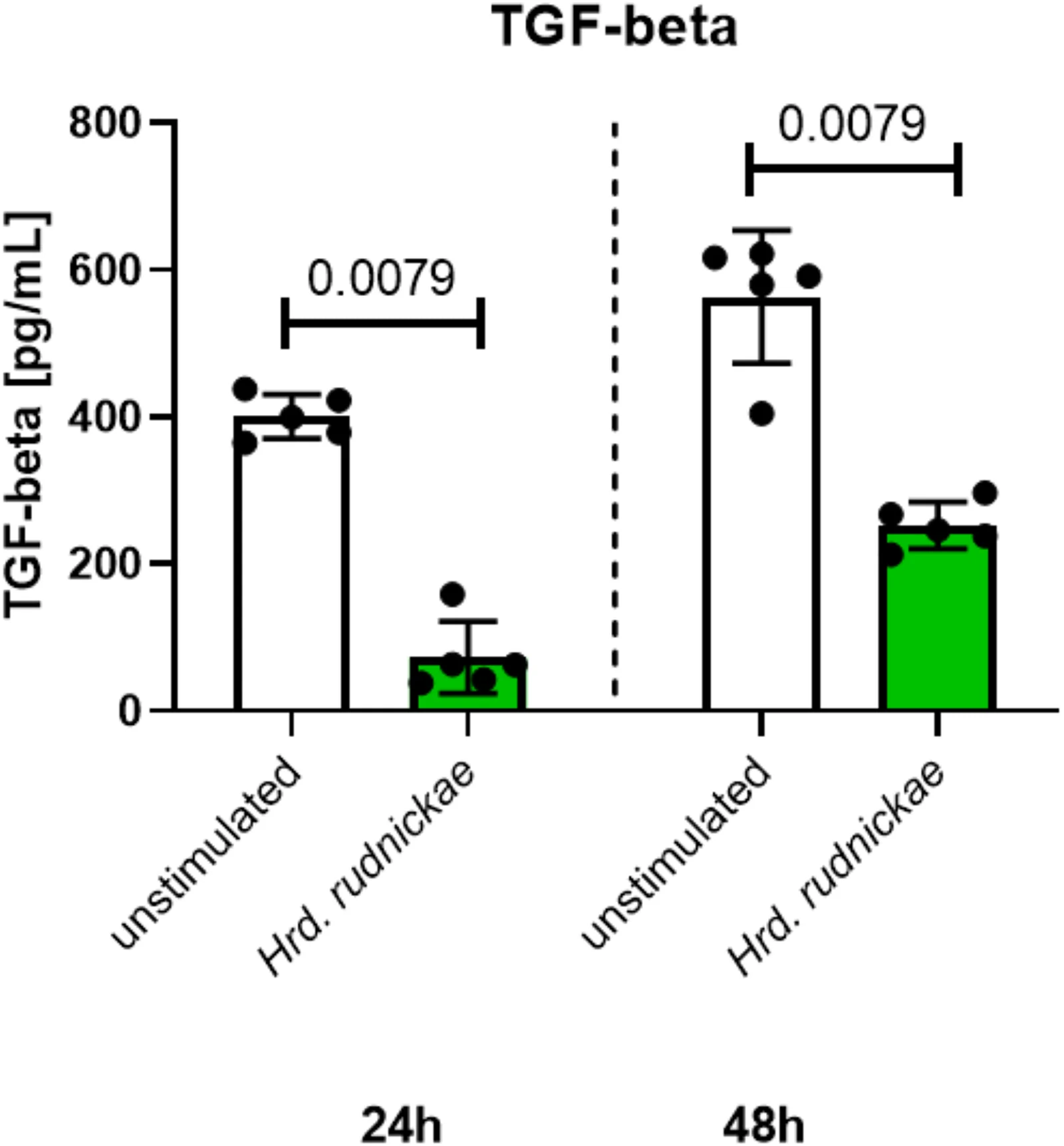
TGF-beta secretion by halophile-stimulated epithelial cells. Calu-3 epithelial cells were either stimulated with *Hrd. rudnickae* or left unstimulated for 24 and 48 hours. The cell culture medium was collected and the level of TGF-beta secretion by the stimulated Calu-3 cells was measured in duplicate by ELISA. Data are presented as means ± SD, while the dots represent individual results. n = 5. Significant p-values are shown on the graph.

### 3.3 The wound healing of the Calu-3 monolayer is induced by *Hrd. rudnickae*

To analyse the potential of halophilic archaea to improve the healing of mechanical disruption to the epithelial barrier, Calu-3 cells were physically damaged using AccuWound 96 tool (ACEA Biosciences, USA) and stimulated with *Hrd. rudnickae*. The migration process was observed at 2-hour intervals using xCELLigence RTCA eSight (**Supplementary Figure 1**) for up to 72 hours. It was observed that Calu-3 cells stimulated with *Hrd. rudnickae* exhibited significantly faster wound coverage after 24, 48 and 72 hours than unstimulated Calu-3 cells (**Figure 3**). Continuous exposure to halophilic archaea led to a time-dependent elevation in Confluence Percentage Area Within the Wound [%]. At 24 h, unstimulated cells reached 21.35 ± 2.41% versus 27.96 ± 2.11% in *Hrd*. *rudnickae*–stimulated cell cultures, an absolute difference of +6.61 percentage points (pp), corresponding to +30.96% relative to control with *p = 0.0086*. At 48 h, control and stimulated cultures measured 29.91 ± 3.32% and 38.15 ± 1.36%, respectively (Δ = +8.23 pp; +27.53%; *p = 0.0014*). At 72 h, values were 34.05 ± 4.18% for control and 41.89 ± 0.62% for stimulated cultures (Δ = +7.85 pp; +23.05%; *p = 0.0021*). Across the three time points, the mean ± SD for each condition and the corresponding absolute (pp) and relative (%) differences are reported to facilitate direct comparison of closure extent between groups. Across all time points, the treated condition consistently exceeded unstimulated control by approximately 6.6–8.2 pp, with the largest relative advantage early in the assay that progressively declined as monolayers approached higher confluence.

**Figure 3:**
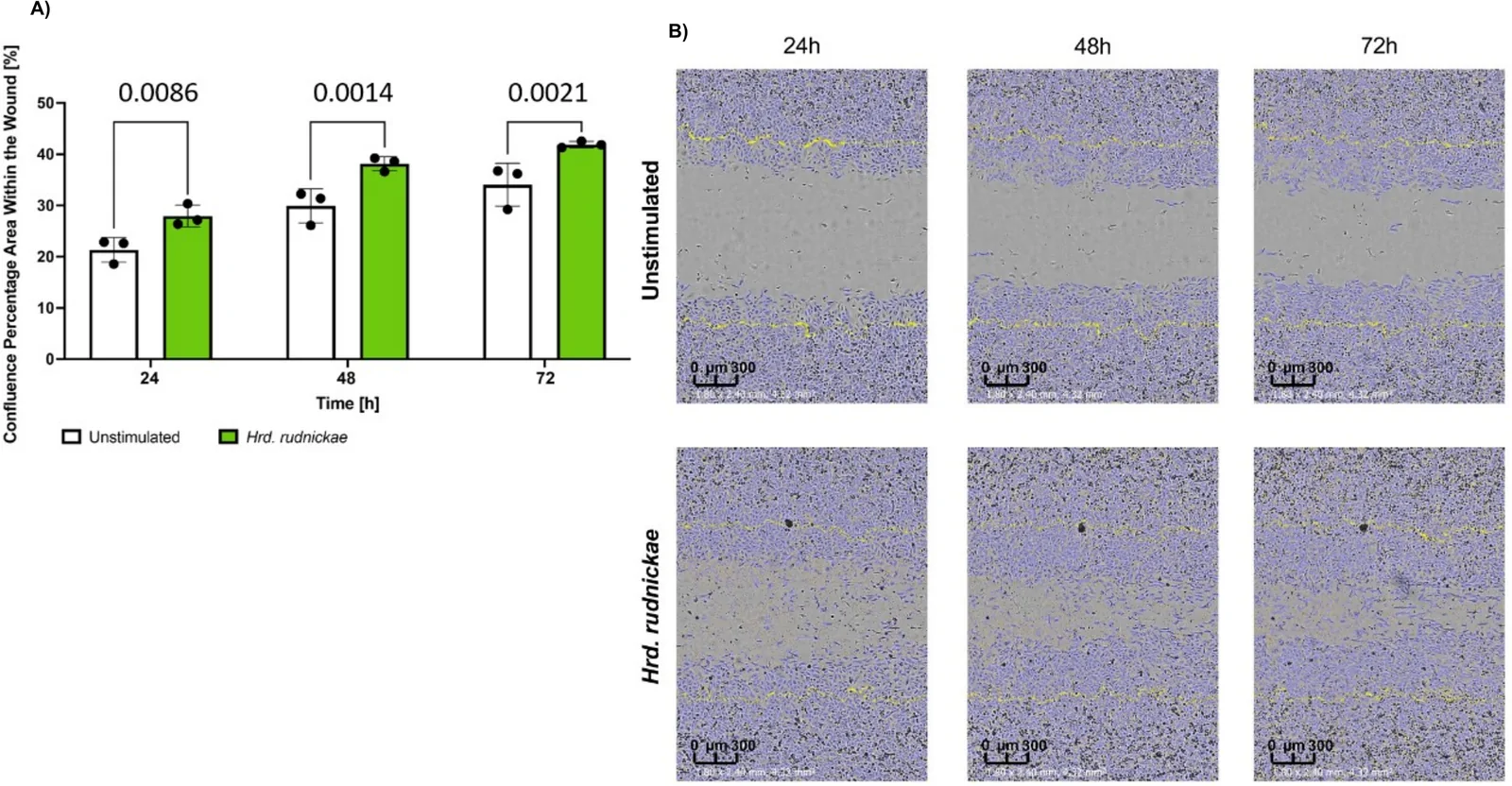
The migration effectiveness of human epithelial Calu-3 cells stimulated with *Hrd. rudnickae* assessed using a wound-healing assay. The Calu-3 cells were grown to confluence prior to the experiments. A wound was made in the monolayer and the medium, or the medium containing *Hrd. rudnickae*, was added. The wound area was measured every two hours. **(A)** The graphs show the percentage confluence within the wound at 24, 48 and 72 hours. Data are presented as means ± SD, while the dots represent individual results (n = 3). Significant p-values are shown on the graph. **(B)** Representative image of the wound healing process in unstimulated or *Hrd. rudnickae*-stimulated Calu-3 cells. The yellow borders indicate the wound area at 0 hours.

### 3.4 Halophilic archaea increase the fluorescence intensity of the ZO-1 protein

The effect of *Hrd. rudnickae* on the Calu-3 tight junction was determined by immunofluorescence staining of the tight junction protein ZO-1 followed by confocal microscopy. Calu-3 monolayers were cultured in µ-Slide chambers (ibidi) to allow direct staining on the imaging surface. Unstimulated cells served as the negative control. After monolayer formation, cells were stimulated with *Hrd. rudnickae* or IFN-gamma (50 ng/mL) as a disrupting factor. *Hrd. rudnickae*-stimulated Calu-3 cells showed a significantly higher ZO-1 fluorescence signal at 48 h (*p = 0.0152*) and 72 h (*p = 0.0043*) compared to unstimulated cells (**Figure 4**). Furthermore, ZO-1 fluorescence was significantly higher in *Hrd. rudnickae*- versus IFN-gamma-stimulated cells at 24 h *(p = 0.0006*), 48 h (*p = 0.0022*), and 72 h (*p = 0.0022*) (**Figure 4**). IFN-gamma, as a disrupting factor, led to a significantly lower ZO-1 signal at 24 h (*p = 0.0041*), 48 h (*p = 0.0411*), and 72 h (*p = 0.0022*) compared with unstimulated cells (**Figure 4**).

**Figure 4:**
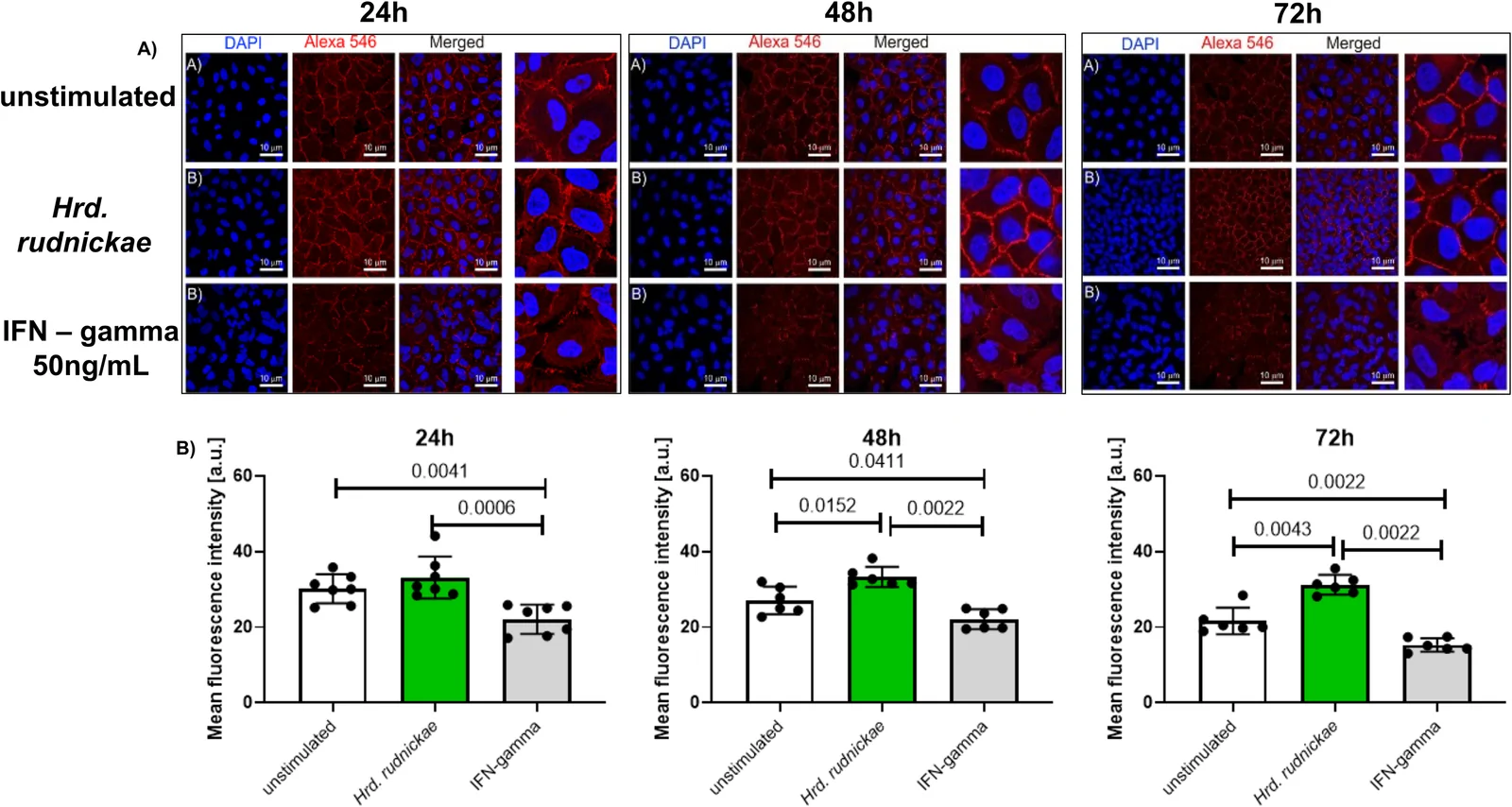
Increase in ZO-1 fluorescence of Calu-3 cells in response to *Hrd. rudnickae* stimulation. Unstimulated Calu-3 cells, as well as cells stimulated with *Hrd. rudnickae* or IFN-gamma as a disrupting factor, were stained and observed using confocal microscopy. **(A)** A representative image of Calu-3 cells stained with ZO-1 monoclonal antibody with Alexa Fluor™ 594 (red) and DAPI (blue) after 24, 48, and 72 hours of stimulation with *Hrd. rudnickae* or IFN-gamma (disrupting factor), or left unstimulated. **(B)** Graphs showing the mean fluorescence intensity of the ZO-1 protein in Calu-3 cells that were either unstimulated or stimulated with *Hrd. rudnickae* or IFN-gamma at 24, 48, or 72 hours. Data are presented as means ± SD, while the dots represent individual results. n = 7. Significant p-values are shown on the graph. a.u. – arbitrary units.

### 3.5 *Hrd. rudnickae* reduced FITC-dextran flux in Calu-3 ALI culture

To determine the tightness of the epithelial barrier, TEER measurements and paracellular flux of FITC-dextran were performed. The structure of the Calu-3 ALI culture on the insert membrane was visualised using phalloidin staining of the cells’ cytoskeleton (**Figures 5A, B**, **Supplementary Figure 2**). TEER measurements showed significantly lower values in the Calu-3 ALI culture stimulated with IFN-gamma (50 ng/mL) cells after 48 hours (*p = 0.0433*) and 72 hours (*p = 0.0152*) of stimulation compared with unstimulated cells at time 0h. No significant changes in TEER values were observed between unstimulated and *Hrd. rudnickae*-stimulated Calu-3 ALI cultures (**Figure 5C**).

**Figure 5:**
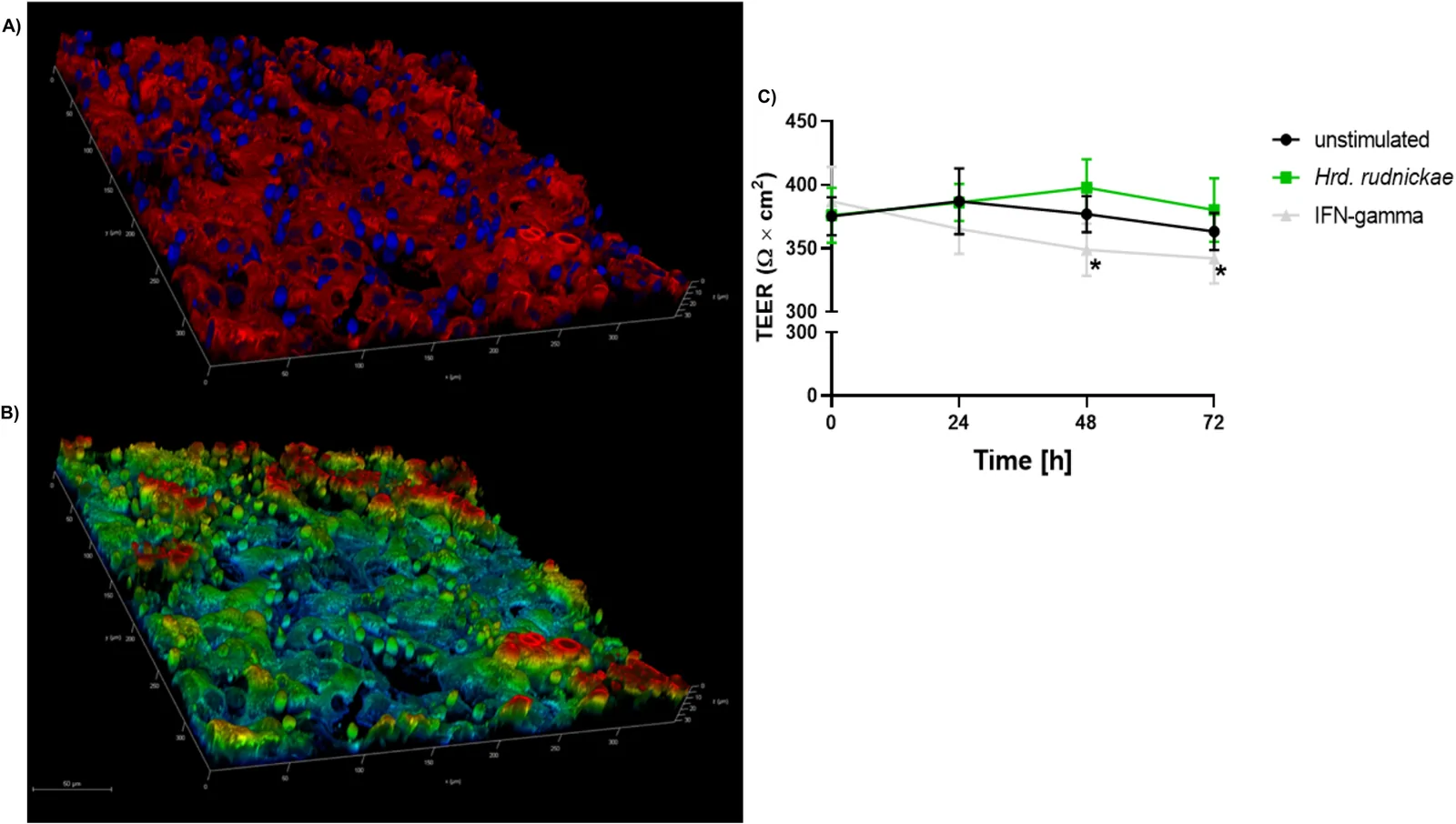
Structure and TEER measurement of Calu-3 ALI culture. Calu-3 cells in air–liquid interface (ALI) culture were stimulated with *Hrd. rudnickae* or IFN-gamma (disrupting factor) or left unstimulated (negative control) for up to 72h. **(A, B)** Confocal microscopy images of unstimulated Calu-3 cells cultured under ALI conditions for 72h. Nuclei were stained with DAPI (blue) and phalloidin–Texas Red (red). **(A)** Surface projection images; **(B)** Topographic scans of the preparations; **(C)** TEER values of Calu-3 ALI culture in response to stimulation. Data are presented as means ± SD; n=6; *p ≤ 0.05, *represents significant differences between certain timepoint and time 0 hours.

To examine the integrity of the epithelial barrier, a FITC-dextran assay was performed. *Hrd. rudnickae*-stimulated Calu-3 cells showed significantly lower fluorescence of FITC-dextran in basolateral c-MEM samples at 48 hours (*p = 0.0022*), but not at 72 hours, after stimulation (**Figure 6**) compared to unstimulated cells. Additionally, stimulation of Calu-3 cells with IFN-gamma (50 ng/mL) showed significantly higher FITC-dextran fluorescence in basolateral c-MEM samples at 48 hours (*p = 0.0022*) and 72 hours (*p = 0.0022*) compared to Calu-3 cells stimulated with *Hrd. rudnickae* (**Figure 6**). Finally, stimulation of the Calu-3 ALI culture with IFN-gamma (50 ng/mL) resulted in increased cellular barrier leakage, as evidenced by significantly higher FITC-dextran fluorescence in the basolateral c-MEM samples at 48 *(p = 0.0173*) and 72 hours (*p = 0.0022*) post-stimulation compared to unstimulated cells (**Figure 6**).

**Figure 6:**
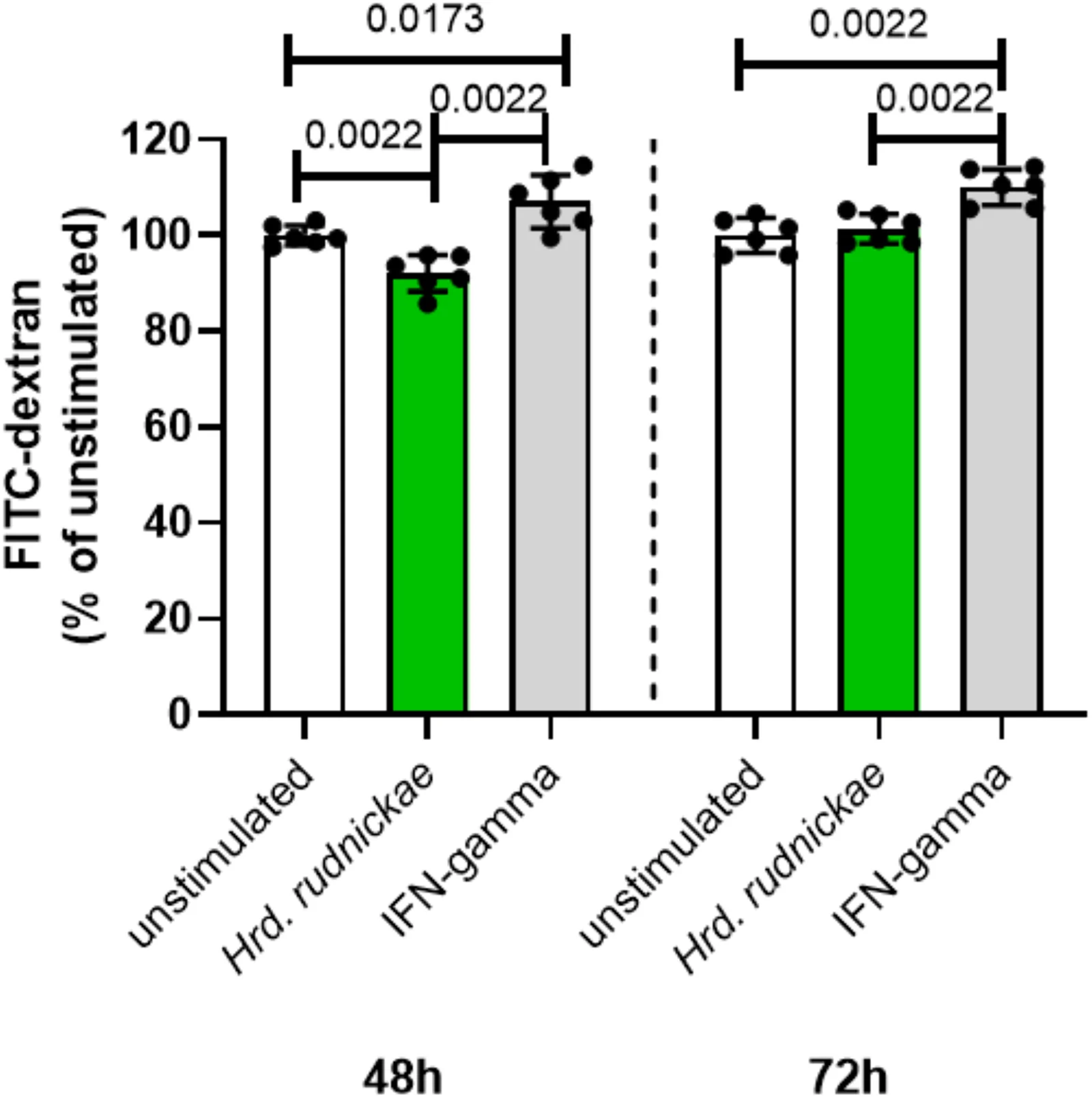
Paracellular FITC-dextran flux of Calu-3 ALI culture. Calu-3 cells in air–liquid interface (ALI) culture were stimulated with *Hrd. rudnickae* or IFN-gamma (positive control) or left unstimulated (negative control) for 48h and 72h. Paracellular flux of FITC-dextran through the Calu-3 epithelial barrier in response to stimulation was analysed. Data are presented as means ± SD, while the dots represent individual results. n=6 Significant p-values are shown on the graph.

## 4 Discussion

To the best of our knowledge, our study is the first attempt to investigate the interaction between halophilic archaea and the epithelium of the upper respiratory tract. In this study, we used a model of upper respiratory tract epithelial cells, which constitute the first line of defence against microorganisms present in the environment. In our recent papers, we demonstrated the presence of halophilic archaea in the air of the Bochnia Salt Mine (Puławska et al., 2025, 2026). The presence of airborne halophilic microorganisms in the salt mine air may indicate their potential to interact with the epithelial cells of the upper respiratory tract in patients and tourists visiting the salt mine. In this study we demonstrated that the stimulation of Calu-3 cells with the halophilic archaea *Hrd. rudnickae* is not cytotoxic or genotoxic to the cells, reduces the production of TGF-beta, accelerates epithelial cell migration and proliferation, strengthens tight junctions between epithelial cells (as represented by increased fluorescence intensity of ZO-1) and, in the ALI model, enhances the epithelial barrier manifested by the reduced FITC-dextran permeability with no changes in TEER values.

Attempts to use biological substances to strengthen the epithelial barrier have been made for a long time. Bacterial strains such as *Akkermansia muciniphila* (Chelakkot et al., 2018), *Lactobacillus plantarum* MB452 (Anderson et al., 2010), and various *Bifidobacterium bifidum* strains (Hsieh et al., 2015) have been found to improve the integrity of the intestinal barrier in mice (Chelakkot et al., 2018), while in the Caco-2 model they have been shown to upregulate the expression of tight junction genes (Anderson et al., 2010) and increased TEER values (Hsieh et al., 2015). In the case of upper respiratory tract epithelial cells, DiGuilio et al. (2023) used a monolayer of Calu-3 cells stimulated with quercetin, a flavonoid commonly found in fruits and vegetables, and demonstrated a positive effect of strengthening the epithelial barrier (DiGuilio et al., 2023). Martens et al. (2018b) as well as Kleniewska and Pawliczak (Kleniewska and Pawliczak, 2024) who discuss the impact of probiotics like *Lactobacillus rhamnosus* GG, *Streptococcus thermophiles* ATCC19258, *Escherichia coli* Nissle 1917, *Lacticaseibacillus paracasei* or *Bifidobacterium infantis* on respiratory diseases including allergic rhinitis, chronic rhinosinusitis as well as asthma and suggested the impact of probiotic administration (intranasal or intratracheal) to strengthen the respiratory tract epithelial barrier.

In the context of archaea domain the studies have focused so far mainly on the impact of methanogenic archaea, like *Methanobrevibacter smithii* or *Methanosphaera stadtmanae* (Bang et al., 2014; Blais Lecours et al., 2011; Weinberger et al., 2025). Blais Lecours et al. (2011) used a murine model, where methanogenic archaeal strains administrated intranasally to the mice resulted in significant histological changes to the lungs, immune cell infiltration (mainly macrophages, lymphocytes, and myeloid dendritic cells), and dose-dependent production of antigen-specific IgG in plasma (Blais Lecours et al., 2011). However, the authors did not examine the impact of methanogenic archaea on the epithelial barrier structure.

In the study by Bang et al. (2014) *M. stadtmanae* and *M. smithii* were used to stimulate the Caco-2/BBe intestinal epithelial cell line. However, Caco-2/BBe cells did not recognized methanogens, as there was no production of the IL-8 and no changes in the transcription of genes encoding TNF-α, IL-8, human beta defensin 1 (HBD1), HBD4, human defensin 6 (HD6) and human cathelicidin LL37 (Bang et al., 2014), were noted.

The study by Weinberger et al. (2025) demonstrated that stimulating the HT-29 cell line (intestinal epithelial cells) with extracellular vesicles derived from methanogenic archaea (*M. smithii* ALI, *M. intestini*, *M. smithii* GRAZ-2 and *M. stadtmanae*) resulted in high CX3CL1 production. However, high IL-8 production was observed only in cells stimulated with extracellular vesicles derived from *M. intestini* (Weinberger et al., 2025). These results suggested a largely similar response to methanogenic archaea, with high CX3CL1 production resulting from stimulation by methanogens, as well as certain species differences, such as IL-8 production only resulting from stimulation by *M. intestini* (Weinberger et al., 2025). Furthermore, the stimulation of HT-29 and THP-1 cells with extracellular vesicles from methanogenic archaea was not cytotoxic, consistent with our previous findings regarding halophilic archaea (Krawczyk et al., 2025).

Our research has shown that the stimulation of Calu-3 cells with *Hrd. rudnickae* did not induce significant changes in cytotoxicity or genotoxicity tests what was observed in the CCK-8 or comet assays up to 48 hours of culture. The lack of cytotoxic and genotoxic effects of *Hrd. rudnickae* on the Calu-3 cell line is consistent with the results of previous research, including the lack of a cytotoxic effect of halophilic archaea on the A549 cell line, which derives from the human lung carcinoma (Kowalewicz-Kulbat et al., 2023).

We have previously demonstrated that *Hrd. rudnickae* as well as *Natrinema salaciae* induced the production of cytokines by dendritic cells and by co-cultures of DCs with autologous CD4^+^ T lymphocytes (Krawczyk et al., 2022). It was observed that DCs stimulated with halophilic archaea secreted significantly more IL-12p40, IL-10 and TNF-alpha, with no changes in IL-23 production (Krawczyk et al., 2022). At the same time, DCs co-cultured with CD4^+^ T cells secreted significantly more IFN-gamma and IL-13 when stimulated with halophilic archaea than unstimulated cells (Krawczyk et al., 2022). We have observed that *Hrd. rudnickae* stimulated the Calu-3 cells to produce TGF-beta, which is a multifunctional cytokine playing a role in various processes, including cell proliferation, cell differentiation, embryonic development, wound healing, immune regulation, tissue fibrosis, and tumour development (Deng et al., 2024). TGF-beta has been demonstrated to be involved in the natural process of epithelial-to-mesenchymal transition (EMT) (Thiery et al., 2009). This phenomenon has been observed in various contexts, including embryonic development, gastrulation, and heart development. Additionally, EMT-like processes have been identified as crucial components in the physiological response to injury (Thiery et al., 2009). However, it is important to note that EMT is also involved in pathological processes, including fibrotic diseases, which are associated with impaired TGF-beta signalling. It has been observed that epithelial cells and alveolar macrophages are the primary sources of TGF-beta, a key mediator in the process of fibrosis within the lungs (Deng et al., 2024). Our study has shown that Calu-3 cells stimulated with *Hrd. rudnickae* led to a significant reduction in TGF-beta production. Targeting the TGF-beta signalling cascade, through neutralising antibodies, small-molecule TGF-beta inhibitors, ligand traps, antisense oligonucleotides, and vaccines, is currently being tested in pre-clinical and clinical trials with the aim of curing patients with cancer and fibrosis (Peng et al., 2022). Although TGF-beta production by Calu-3 cells and a reduction in TGF-beta production by Calu-3 cells as a result of *Hrd. rudnickae* stimulation was observed, the cells themselves appear to be unaffected by TGF-beta stimulation as other authors have observed. In the study by Buckley et al (Buckley et al., 2010), it was observed that Calu-3 cells did not change their morphology as a result of TGF-beta stimulation, did not show significant changes in the expression of E-cadherin and vimentin, and the rate of wound healing (Buckley et al., 2010). In another study, epithelial cell line, A549, responded to TGF-beta by undergoing both morphological and biochemical changes consistent with those seen in EMT. This highlights the varied response of cell lines to TGF-beta and indicates that the results obtained cannot be directly extrapolated to primary cells (Buckley et al., 2010).

In our work, the effect of halophilic archaea on the migration and/or proliferation of epithelial cells has been checked using a scratch assay. A mechanically damaged Calu-3 monolayer was stimulated with *Hrd. rudnickae*, which resulted in significantly faster wound healing than in unstimulated cells. Considering the previously described publication by Buckley et al (Buckley et al., 2010), the proliferation and/or migration of Calu-3 cells appear to be independent of the autocrine action of TGF-beta and changes in the production of this cytokine. The mechanism by which halophilic archaea support to accelerate wound healing remains unexplored. It may be related to the presence of carotenoids, which are an important metabolite of many halophilic archaeal species (Rodrigo-Baños et al., 2021). Interestingly, carotenoids present in *Kocuria* sp. RAM1, marine pigmented bacteria, have shown a similar effect, accelerating wound healing in HSF (human skin fibroblasts) and OEC (oral epithelial cells) cell lines (Metwally et al., 2022). However, the possible impact of carotenoids produced by *Hrd. rudnickae* on the activity of Calu-3 cell line requires further studies and remains at this stage speculative.

The primary role of tight junctions (TJs) is to form a paracellular barrier that restricts the passive diffusion of various substances, including ions, macromolecules and pathogens, across the paracellular space. In doing so, TJs preserve the electrochemical and osmotic gradients generated by active transcellular transport (Citi et al., 2024). Vermette et al. (2018) indicated that various diseases cause profound changes to the structure of TJs, which consequently results in a decrease in the integrity of both epithelial and endothelial barriers. Furthermore, TJ breakdown markers correlate with clinically relevant outcomes in many disease states at clinically relevant time points (Citi et al., 2024). Damage to TJs results in the development of many inflammatory diseases, including atopic dermatitis, asthma, inflammatory bowel disease, allergic rhinitis (Akdis, 2021).

Our research has shown that halophilic archaea *Hrd. rudnickae* have the potential to improve the epithelial barrier of the Calu-3 cell line. In a model of Calu-3 cells, cultured in a monolayer system, and stimulated with *Hrd. rudnickae*, we noticed a significantly increased fluorescence intensity of ZO-1 compared to unstimulated cells. ZO-1 is capable of binding to and interacting with a wide variety of proteins, such as claudins, occludin, ZO-2, ZO-3, cingulin, afadin, JAM-A, α-catenin, shroom2, F-actin and angulin, among others. In epithelial cell cultures, the simultaneous loss of ZO-1 and ZO-2 impairs the recruitment of claudins to tight junctions, resulting in a significant weakening of barrier function. Furthermore, epithelial cells lacking ZO-1 exhibit a significant impairment in initiating effective proliferation during wound healing (Kuo et al., 2022). In our research, in the air-liquid interface (ALI) model, it was demonstrated that stimulation with *Hrd. rudnickae* improved epithelial barrier by significantly reduction of FITC-dextran permeability. Interestingly, this was only observed during 48-hour stimulation and not during 72-hour stimulation. Further research into the role of halophilic archaea should consider a wider range of time points. However, in our study no statistically significant changes in TEER values were observed. IFN-gamma, used as a disruptive factor, caused a significant decrease in fluorescence intensity of ZO-1, lower TEER values and higher FITC-dextran permeability which is consistent with data in the literature (Petecchia et al., 2012; Utech et al., 2005).

While our results provide evidence consistent with the hypothesis that airborne halophilic archaea may enhance the epithelial barrier integrity in Calu-3 cell line, several limitations of the present study should be acknowledged. In particular our study is lacking of knowledge about the mechanism involved in the interaction of halophilic archaea with epithelial cells. It remains unknown whether halophilic archaea, similarly to the methanogenic archaea, can activate the immune system through their RNA and the TLR8-dependent NLRP3 inflammasome activation pathway (Vierbuchen et al., 2017), thus this requires further study. Another limitation is that our study has focused on Calu-3 cell line and we have not investigated the effect of halophilic archaea on primary upper respiratory tract epithelial cells. As Martens et al. (2018a) noted that Calu-3 cells may differ from freshly isolated primary nasal epithelial cells, thus it would be interesting to extend the study on primary epithelial cells in the future research.

## Conclusions

To summarise, the present study demonstrates that stimulation of Calu-3 cells with halophilic archaea *Hrd. rudnickae* did not induce toxicity or genotoxicity in epithelial cells, improved physical wound healing process and reduced the production of TGF-beta by Calu-3 cells. Moreover, halophilic archaea strengthened the epithelial barrier what was indicated by increased fluorescence intensity of ZO-1 and reduced FITC-dextran permeability. Research on halophilic archaea in the context of the epithelial barrier is still in its infancy. Further research is needed to identify the mechanism involved in the interaction of halophilic archaea with the epithelial cells. Nevertheless, we believe that halophilic archaea may have potential applications in the future in the treatment of diseases characterised by a damaged epithelial barrier.

## Data Availability

The raw data supporting the conclusions of this article will be made available by the authors, without undue reservation.

## Supplementary material

Supplementary Figure 1 Calu-3 cells were grown in a monolayer prior to stimulation, after which they were stimulated with *Hrd. rudnickae* or left unstimulated (negative control). The scratch was made at time 0. Photographs were taken every 2 hours. The yellow lines represent the wound boundaries.

Supplementary Figure 2 Confocal microscopy image of Calu-3 ALI culture. Calu-3 cells stained with phalloidin-texas red (cytoskeletal staining) and DAPI (cell nucleus).
